# The Diseases of Females: Including Those of Pregnancy and Childbed

**Published:** 1843-02-18

**Authors:** 


*The Diseases of Females: Including those of Pregnancy and Childbed.* By FLEETWOOD CHURCHILL, M. D., etc., etc. Second American Edition. With Notes. By ROBERT M. HUSTON, M. D., Professor of Materia Medica and Therapeutics in the Jefferson Medical College, etc., etc. Philadelphia: 1843. 8vo. pp. 575.

This work comprises Dr. Churchill's Treatise on the Diseases of Females, with that on the Diseases of Pregnancy and Childbed. Its publication is an acceptable service to the profession, supplying a want universally felt. Though defective in many points, and more characterized by plodding industry than original talent, it is, perhaps, the best and most complete book on the subject in the language, and we can cordially recommend it to the student and practitioner. The additions of Professor Huston are chiefly to the chapters on Chlorosis, Uterine Leucorrhœa, Prolapsus Uteri, Ovaritis, Convulsions, Puerperal Fever, and Rupture of the Uterus; and though not very extensive, supply some omissions on the part of the author, and render the work more complete. The remarks of the Editor on the nature and treatment of Puerperal Fever are just and sound, and his caution against the indiscriminate employment of bleeding in this malady, when epidemic, is timely and judicious. Even where the local disease would seem to justify the employment of depletion, the constitutional condition frequently forbids its use.

